# The Impact of Metal Nanoparticles on Female Reproductive System: Risks and Opportunities

**DOI:** 10.3390/ijerph192113748

**Published:** 2022-10-22

**Authors:** Massimo Aloisi, Gianna Rossi, Sabrina Colafarina, Maurizio Guido, Sandra Cecconi, Anna M. G. Poma

**Affiliations:** Department of Life, Health and Environmental Sciences, University of L’Aquila, 67100 L’Aquila, Italy

**Keywords:** nanoparticles, reprotoxicity, cryopreservation, ovarian cancer

## Abstract

Humans have always been exposed to tiny particles via dust storms, volcanic ash, and other natural processes, and our bodily systems are well adapted to protect us from these potentially harmful external agents. However, technological advancement has dramatically increased the production of nanometer-sized particles or nanoparticles (NPs), and many epidemiological studies have confirmed a correlation between NP exposure and the onset of cardiovascular diseases and various cancers. Among the adverse effects on human health, in recent years, potential hazards of nanomaterials on female reproductive organs have received increasing concern. Several animal and human studies have shown that NPs can translocate to the ovary, uterus, and placenta, thus negatively impacting female reproductive potential and fetal health. However, NPs are increasingly being used for therapeutic purposes as tools capable of modifying the natural history of degenerative diseases. Here we briefly summarize the toxic effects of few but widely diffused NPs on female fertility and also the use of nanotechnologies as a new molecular approach for either specific pathological conditions, such as ovarian cancer and infertility, or the cryopreservation of gametes and embryos.

## 1. Introduction to Nanoparticles

In European legislation, a nanomaterial is defined as “an insoluble or bio-persistent and intentionally manufactured material with one or more external dimensions, or an internal structure, on the scale from 1 to ≤100 nm” [1,2]. The most important parameters for defining a nanomaterial are its size and physicochemical properties. The mode of formation of nanomaterials can be of two types: “top-down” or “bottom-up.” In the first case, they are intentionally synthesized for specific purposes or result from the random degradation of materials dispersed in the environment resulting in the formation of pollutants. In both, they are obtained by the degradation of a starting material. In the second case, they result from chemical processes and not from bulk starting materials [3]. Understanding the size and chemical characteristics of the nanomaterial is crucial, because kinetic properties, which is a set of parameters designed to estimate how much material is absorbed and its path within a living organism [4], also depend on these. Considering the dimensions as the main characteristics, we can define nanoparticles (NPs) as “nano-objects with three external nanoscale dimensions”. The terms nanorod or nanoplate are employed, instead of NPs, when the longest and the shortest axis lengths of a nano-object are different [5].

NP spread in the environment is very relevant. Indeed, we can distinguish natural nanoparticles from those man-made and then dispersed [6,7]. Atmospheric NPs can originate from forest fires and volcanic eruptions, and populations living near areas with a high frequency of these phenomena have higher hospitalization rates and more frequent respiratory problems [7,8,9]. NPs released from anthropogenic activities are concentrated mainly near urban areas with a prevalence of those smaller than 50 nm [10], and major pollutants include vehicle emissions and tire-wear particles [11,12,13]. Also, occupational hazards contribute to their formation, as the most common chemically induced occupational disease is silicosis [14]. Cigarette smoke contains many NPs interacting with the gas phase and together giving a synergistic effect that could amplify the adverse effects of each individual component [15,16,17]. 

Other NPs gaining significance as a pollutant are nanoplastics [18]. The toxicity of plastic wastes was understood as early as 1969, but it was not until the development of new technologies that their degradation products were also identified [19,20]. They are debris of different shapes and sizes that tend to remain in suspension given their small size [21]. Being lipophilic, they accumulate mainly in fatty tissues, and by biomagnification, their concentrations increase as one moves up the trophic chain of the ecological niche considered [22]. 

Depending on its composition, an NP can be lipophilic or hydrophilic: the former is able to cross cell membranes by diffusion, while the latter needs active transport [23]. NPs can be chemically modified on their surface by varying their charge to increase properties either in a targeted manner (e.g., to aid adsorption when they act as carriers for drugs), or randomly (e.g., when other pollutant molecules dispersed in the environment are adsorbed on them) [24]. Uncharged NPs tend to bind with serum proteins and, when incubated with cells, their uptake is mediated by specific receptors [25,26]. In general, positively charged NPs are taken up by endocytosis much more often than negatively charged ones because of the presence of phospholipidic molecules [27,28,29]. In addition, NPs can be bound to bioconjugates, specific biological molecules recognized by cells receptors [30]. The main mechanisms of NP endocytosis are clathrin-mediated, macropinocytosis and phagocytosis [31], which can occur independently of cargo size [32,33]. 

**Table 1:** Nanoparticles (NPs) widely used in cosmetics and food.

	NPs	Where	Dimensions	References
Cosmetics				
	TiO_2_	Sunscreens, toothpastes	200–400 nm	[34,35,36]
	ZnO	Sunscreens, toothpastes	10–200 nm	[37,38,39]
	Sb, As, Cd, Pb, Cr	Eye shadow, lipstick, soap, eye shadow, body creams	/	[40]
	Liposomes	Skin creams and drugs, soap	Variable	[41]
	Nanoemulsions	Hair care products	50–200 nm	[42]
	Micro- and Nanoplastics	Facial scrubs, toothpastes, nail products, shower gel	10–100 nm/0.1–1 μm	[43]
Food				
	Nanocellulose	Milk products, salad dressing, ice cream	5–100 nm	[44]
	Sufactantcoated NPs	Beverages, ice cream, margarine	1–100 nm	[45]
	SiO_2_		1–100 nm	[46]
	Cu/CuO	food packaging		[47]
	Nanopolyethylene	Food packaging	1–100 nm	[48]

After entering in the circulatory system via various items (food/oral ingestion, inhalation, skin penetration), they can exert their negative toxic effects on cells by increasing the production of reactive oxygen species (ROS), damaging DNA and mitochondria and even inducing cell death [49]. Table 1 shows the most common and everyday products in which NPs can be found. In cosmetic products they are used primarily to contribute to the desired effect, especially antiaging, and secondarily as preservatives, and for these reasons have a larger size. In food, they are added for antibacterial purpose and to increase shelf life. Also, the possibility that NPs can result from the degradation of transport coatings and subsequent passage into food cannot be excluded. A link seems to exist between NPs and cancer: for example, iron-rich NPs from traffic pollution are likely a plausible cause of brain cancer, while a significant association between wildfires and the incidence of specific cancer outcomes (as lung, brain and some haematological cancers) has been recently proposed [50,51]. 

With regard to metallic NPs, their effects depend mainly on their composition, morphology, size and crystal structure. They are characterized by a metal core composed of inorganic metal or metal oxide usually covered with a shell formed by organic, inorganic material or metal oxide. The most widely used metallic NPs in medicine, food, agriculture and industry include Ag, Au, ZnO, TiO_2_, CuO, CeO_2_ and FeO. Among these, TiO_2_ NPs are particularly popular in biomedical applications, being employed for the fabrication of biomedical devices and for bioimaging [52]. Considering the breadth of applications to date, little is known about their toxicity except for a few metal NPs used in biomedical fields [53].

Since 2015, the European Commission has launched a series of proposals with the aim of reducing their use both to safeguard human health and the environment [54]. In 2021, the European Food Safety Authority (EFSA) produced a summary document on the main procedures for determining NP counts in food [55]. For example, the use of TiO_2_NPs as a food coloring and flavoring agent is no longer considered safe because of its effects on DNA. Despite its low bioavailability, it can accumulate in the body reaching high chronic concentrations especially in long-living animals and breeding animals [56]. To date, it remains usable in medicinal products at depletion [57,58]. The presence of TiO_2_NPs in cosmetics as a preservative and colorant has been reduced to 25% in masks and hair products, a value considered safe without prejudice to its carcinogenic capacity by inhalation [59]. Other NPs, including those listed in Table 1, are still under observation.

The reproductive toxicity of these NPs is becoming a public health problem due to the use of NP-containing consumer products and occupational exposure. Both male and female reproductive organs are vulnerable, but in females the presence of NPs may compromise fertility and eventual fetal development more dramatically due to the fixed and not renewable number of oocytes present at birth in the mammalian ovaries. Furthermore, NP accumulation seems to be strongly conditioned by menstrual cycle, as demonstrated by the 2- fold increase in ovarian and uterine NP content occurring during mouse ovulation in comparison with the other stages of estrus cycle [60]. In this review we will discuss not only of the toxic effects of selected NPs on female reproductive organs but also of their importance for fertility preservation. 

## 2. Path of NPS Access into Cells of Female Reproductive System

Since NP intake via skin is considered to be below detection level, ingestion and inhalation represent the major route by which NPs are transferred to organs and cells, as confirmed by their presence in the circulatory and lymphatic systems as well as urine [61]. Since NPs are able to cross biological barriers in a way strongly conditioned by physical characteristics (size, shape, etc.), they can be deposited in the target organs reaching cytoplasm and mitochondria. In testis, NPs can cross the blood–testis barrier which can be completely harmed due to increased ROS production [62], while they can accumulate in the cells of female reproductive organs by mechanisms not completely understood yet, but capable of inducing significant increase of ROS levels. Even if a key point is to define if and how NPs can be internalized into oocytes, their storage in the somatic compartment of the ovary results in a dramatic impairment of oocyte developmental competence, which is the ability to be successfully fertilized and to form a blastocyst. This property is acquired in a stepwise manner during folliculogenesis, and is strongly conditioned by the maintenance of bidirectional communications between the somatic and germinal compartment [63]. Below, we will report recent data focusing on the reproductive toxic effects and utilization for therapeutic purposes of some of the most-used NPs. 

## 3. NP-Induced Toxicity in Ovary and Oocytes 

### 3.1. Zinc Oxide Nanoparticles (ZnONPs or nZnO) and Titanium Dioxide Nanoparticle (TiO_2_NPs or nTiO_2_) 

Both NPs are commonly used in a wide range of products, from paints to cosmetics. nTiO_2_ is produced in three commercial forms named rutile, brookite and anatase, which is the most utilized [64]. In recent years, several studies have investigated their toxic effects in vivo and in vitro.

#### 3.1.1. In Vivo Exposure

The mechanisms by which TiO_2_NPs can damage ovarian cells have mainly been studied in laboratory animals. Experimental data demonstrated that a long-term administration (5–8 weeks) of TiO_2_NPs (1–10 mg/kg/d) to female mice caused alterations of gonadotropin and sex steroid release, cysts formation and follicular atresia together with significant reduction of pregnancy rate due to extensive ovarian inflammation caused by elevated ROS production [65,66,67]. Interestingly, these NPs can negatively interfere with the TGF beta pathway, which plays important roles in regulating several ovarian functions, including follicle development and maturation [68]. Ovarian mitochondrial damages and the expression level of apoptotic genes increased concomitantly with the accumulation of TiO_2_NPs (1.25–2.5–5 mg/kg for 90 consecutive days) in both the cytoplasm and nuclei of follicle cells [69]. The rise of oxidative stress level and of Cyp17a1 gene expression, together with altered regulation of many (about 288) ovarian genes, account for the decline of fertility and pregnancy rate. In vivo, increased estrogen release and decreased testosterone/progesterone levels have been detected in rodents of various strains treated with increasing concentrations of TiO_2_NPs [70]. Contrasting effects on steroid hormone release have been obtained by Hong and Wang [71] and Zhao et al. [72] following mice exposure to similar concentrations of anatase-nTiO_2_ (2.5–5–10 mg/kg for 30 days), but differences can be ascribed to different treatment times and different mouse strains used in the experiments.

The rapidly expanding production and use of ZnONPs in consumer products (e.g., tooth paste and food packaging) also stimulated studies on this nanomaterial and its ability to alter/disrupt cell functions. nZnO toxicity seems to mainly be due to Zn ion release, although an intrinsic particle toxicity should be considered especially after long-term exposure [73]. In females, reprotoxic effects induced by nZnO (5–10 μg/mL for 24 h) consisted of the alteration of ovarian gene expression, stimulation of apoptosis, interference with Sonic Hedgehog pathway, which has a key role in the regulation of tissue homeostasis, and with steroid hormone synthesis [74,75]. Exposure to nZnO of mouse fetal ovaries (0.2–0.8 mg/kg for two days) resulted in the accumulation of double-strand breaks (DSBs) in pachytene oocytes, with a consequent decrease of ovarian follicle reservoir. Interestingly, ZnO-dependent oocyte damage relies on different mechanisms depending on in vitro or in vivo exposure: in the former, excessive accumulation of DSBs lowered the number of germ cells via apoptosis; in the latter, oocyte selection mechanisms triggered the elimination of ovarian follicle reserve [76].

#### 3.1.2. In Vitro Exposure

The presence during culture of TiO_2_NPs inhibited rat follicle development and oocyte maturation in a size- and concentration-dependent manner [77]. In fact, when rat preantral follicles were cultured for 10 days in the presence of 1 μm or 25 nmTiO_2_ at increasing concentrations (12.5–25–50 μg/mL), follicle development and oocyte maturation were dramatically inhibited only in the presence of 25 nm TiO_2_ at 25 μg/mL or above. The presence of TiO_2_NPs did not hamper the process of cumulus expansion in mice, while a dramatic negative effect was caused by ZnO NM-110 and NM-111 at relatively low concentrations (1 mg/mL). Indeed, experimental data showed that ZnONPs, but not SiO_2_-coated ZnONPs, affected the expression of genes fundamental for the regulation of this process. The negative effect seems to be based either onion release or NP presence per se [78].

Another recent study performed on mouse antral follicles [79] showed that when antral follicles were exposed in vitro to ZnONPs or TiO_2_NPs (5–50 μg/mL for 96 h), the negative impacts on somatic and germ cells were significantly different. First of all, TEM analysis showed the absence of ZnONPs in antral follicular cells, while TiO_2_ NPs were easily detectable in theca, granulosa and cumulus cell cytoplasm as agglomerates enclosed in small vesicles or near mitochondria. Moreover, the presence of invaginations on cell surface confirms that TiO_2_NPs were internalized by micropinocytosis or clathrin-mediated endocytosis. Both NPs had a great impact on cytoskeleton arrangement and trans-zonal projections (TZP) between somatic cells and oocytes, which play a central role in the fine regulation of normal oocyte and follicle development [80]. Surprisingly, the two types of NPs exerted opposite effects on follicle diameters (TiO_2_: increase; ZnO: decrease), but none of them have been found in cultured oocytes that were indirectly damaged because of TZP disruption. Moreover, nZnO toxicity, evidenced by ROS increase, was higher than that caused by nTiO_2_, probably as a result of Zn ion release in culture media. A similar mechanism has been described as occurring also in other cells [80]. The cytotoxic and genotoxic effects of nTiO_2_ and nAl_2_O_3_ were evaluated on Chinese hamster ovarian (CHO-K1) cells. Changes in lysosomal and mitochondrial dehydrogenase activity as well as the formation of perinuclear vesicles in CHO-K1 cells were demonstrated after treatment with both NPs, but none was detectable in the nuclei [81].

### 3.2. Gold and Silver NPs In Vivo and In Vitro

Other metallic NPs can impair follicle survival and ovarian steroidogenesis. Silver nanoparticles (AgNPs) are used for their antimicrobial activity in food, home appliances, water treatment and for disinfecting medical devices [82]. Gold nanoparticles (AuNPs), instead, are a new application in nanomedicine because of the absence of toxic effects at the doses used for drug delivery [83]. However, AuNPs were internalized by cultured buffalo granulosa cells and operated as ovarian endocrine disruptors by perturbing steroidogenesis. In particular, progesterone production was significantly increased, probably as a consequence of increased mitochondria permeability due to particle movement across the cells and other membranes. Such a disturbance of the mitochondrial membranes and homeostasis generates ROS that, in turn, causes oxidative stress and even cell death by stimulating the expression of pro-apoptotic genes [84,85]. Different results were obtained by Tiedemann et al., because in their experiments AuNPs showed (after 46 h) no detrimental effects on porcine cumulus–oocyte complexes regardless of size, surface ligands or concentration [86]. Despite a selective uptake of AuNPs by oocytes, they did not have toxic effects and the authors proposed these particles as a top candidate for biomedical applications.

Other studies evaluated the reproductive risks associated with exposure to widely used AgNPs. Cytotoxic responses include increased levels of ROS production, apoptosis, DNA damage and inflammation, although the molecular and cellular mechanisms by which they can exert adverse effects at the organismal level remain elusive [87]. Porcine cumulus cells can accumulate AgNPs, which caused the alteration of cell proliferation with indirect consequences to oocyte maturation, probably due to ion release [88,89]. In addition, the expression of proliferation-related and apoptosis-related peptides such as cyclin B1 and caspase-3 had been changed in porcine ovarian granulosa cells after AgNPs addition [90]. Similar results were obtained in mice intravenously injected with increasing doses (0–50 mg/mL) of these particles, since they accumulated in a dose-dependent manner in ovaries and induced inflammatory response as well as granulosa cell and theca cell apoptosis. As expected, a significant decrease in the expression levels of genes involved in steroidogenesis and in the maintenance of follicle survival from primordial to antral stage was found [90]. Oocyte damage was also recorded after in vitro exposure to AgNPs. Again, cytoskeletal modifications culminated with retraction of TZPs, which caused the impairment of cumulus expansion and of meiotic resumption in pig cumulus–oocyte complexes exposed during IVM [91].

## 4. Uterus and Placenta Are Targeted by NPs

The presence of NPs in the uterus can induce developmental toxicity and has deleterious consequences to reproductive performance. In in vivo-exposed animals, TiO_2_NPs and AgNPs (0.2, 0.4, or 0.8 µM for 2 h) have been detected in the uterus where increased oxidative stress and apoptosis of connective tissues with consequent alteration of contractile activity were observed [92,93,94].

It is generally accepted that fetuses are more sensitive to environmental toxins than adults [95]. Recent evidence showed that NPs can easily cross the placenta to reach the fetal compartment in a size-dependent manner. In fact, experiments conducted with animal models showed that NPs can negatively impact a fetus in a way strongly conditioned by chemical structure (size, composition, charge, etc.) and by the stage of pregnancy. For example, small silica nSP70 and 35 nm TiO_2_ NPs, administered once every three days from day three of gestation until delivery at 0.02 and 2 mg/Kg, might impair normal fetus development in comparison with higher diameters by causing DNA damage in offspring [96]. These NPs triggered fetal inflammation, apoptosis, and genotoxic and neuronal damage, which resulted in abnormal embryonic development and fetal death [97]. Most studies on placental toxicity have been carried out using laboratory animals, but the existence of several structural differences in placenta organization between rodents and humans has stimulated the use of human cellular models. In pregnant women, NPs may cross the placenta by molecular transport mechanisms that are still poorly understood, although endocytosis and passive transport are the most accredited. Exposure to AgNPs increases the risk of damage to the nervous system of fetuses and also other nano-oxides (as Zn, Fe and Cd oxide) can induce cytotoxic effects and cell death [97].

It is evident that the indirect toxicity of NPs on placental/fetal functions and long-term health requires further exploration, especially because the key steroidogenic role of placenta during pregnancy could be affected by the suspected endocrine disrupting effects of specific nanomaterials used in NP generation [98].

## 5. NP-Induced Genotoxicity

One of the main problems related to nanomaterials is their ability to induce genotoxic, and thus mutagenic and clastogenic, damage on DNA [99]. The damage can be direct, if there is interaction with the genetic material, or indirect, if it is mediated by the formation of chemical species such as ROS [99]. This second mechanism is very important, since NPs are unlikely to reach the nucleus [100]. A recent study on AgNP genotoxicity evidenced positive results based on several in vitro and in vivo tests (in vivo: micronucleus tests, chromosome aberration tests, comet assays; in vitro: mouse lymphoma assays, micronucleus tests, comet assays). Even if AgNPs impaired the DNA repair system and depleted cells of antioxidant molecules, results did not confirm with certainty that the genotoxic effect was produced exclusively by oxidative damage. Therefore, the authors suggested performing a complete genotoxicological assessment of this, and obviously of other NPs, before drawing definitive conclusions on human exposure [101,102]. The use of nanotechnologies for diagnostic and therapeutic purposes based on injections of TiO_2_ and AuNPs in laboratory animals have increased the appearance of new metastatic sites by increasing the gap between blood vessel cells and allowing cancer cells to migrate more easily to new sites, pointing to the need of accurate studies before using NPS to treat cancers [103].

## 6. The Benefits of Nanotechnologies

### 6.1. Preservation of Fertility

The correct application of cryopreservation technologies is fundamental to improve organ/cell shortage for transplantation. Fertility in young women with cancer but also in healthy young women who electively wish to delay childbearing to avoid the detrimental effects of aging on reproductive capacity. Cryopreservation of germ cells requires the use of specific freezing/thawing cocktails, in which different cryoprotectant agents (CPA) and other chemicals are added depending on freezing procedures (slow freezing or vitrification). Due to its rapid procedure, vitrification is now the most-used method to preserve germ cells and embryos.

Unfortunately, the survival rate of vitrified oocytes is still low (about 4%) [104], but recent data showed that the incorporation of specific NPs into CPA solutions could improve outcomes by avoiding recrystallization of vitrification solution during rewarming [105]. Experiments conducted on porcine oocytes arrested at GV stage and vitrified with or without different concentrations (0.01%, 0.02%, 0.05%, 0.1%, 0.5% *w*/*w*) of silicon dioxide (SiO_2_), aluminum oxide (Al_2_O_3_), hydroxy apatite (HA) and TiO_2_NPs (diameter: 20 nm) showed that HA and SiO_2_ are the most biocompatible nanomaterials having a reduced toxicity. In fact, when used at 0.05%, oocyte survival rate and capacity to complete meiosis up to MII stage were higher in comparison with the other NPs tested and control conditions [106]. Similar protective effects were also obtained for vitrified spermatozoa following ZnONP supplementation, and analysis by confocal and high-resolution transmission electron microscopy clearly demonstrated that these NPs did not enter spermatozoa cytoplasm [107]. More recently, Abbasi et al. also showed that Fe_3_O_4_NPs (0.004%, 0.008% and 0.016% *w*/*v*) could protect mouse GV oocytes from cryodamage during vitrification procedures. After IVM and IVF, the quality of frozen/thawed embryos has been found to be positively modulated by presence of these NPs [108].

Another interesting aspect of female fertility preservation in which NPs are starting to use is contrasting ovarian aging. Despite the existence of various strategies, no clinical treatments are currently available to delay ovarian aging. Drug delivery via NPs should be effective in postponing ovarian aging. In fact, studies are currently being pursued to use them in both 3D cultures and tissue engineering, with the purpose of increasing cell proliferation and viability [109].

### 6.2. Ovarian Cancer Therapy

A significant improvement in the safety of NP-delivered drugs in cancer patients has been achieved in recent years to bypass the limitations of chemotherapy. NPs are utilizable for biomedical applications to facilitate the absorption of drugs that can be bound on their surface [110,111]. To date, the most widely used in diagnostics and bioimaging as well as in cancer therapies are AuNPs because they are stable metals with low cytotoxicity, but also gold and carbon nanotubes could be used to treat some forms of cancer, for bioimaging and gene therapy [112].

Recently, the application of RNAi-based therapies has been sharply accelerated because a significant decrease in nonspecific toxicity can be obtained by encapsulating multiple payloads of drugs within nanocarriers and by monitoring si-RNA delivery by noninvasive imaging techniques. Nano-siRNA drugs can be of great help in overcoming the limitations of chemotherapy. Recently, NP-mediated siRNA delivery strategies such as polymeric- and lipid-based systems, rigid nanoparticles and NP coupled with specific ligand systems have been tested. NP-based codelivery of anticancer drugs and siRNA targeting various mechanisms of multi-drug resistance is a cutting-edge strategy for ovarian cancer therapy. Since siRNAs that are negatively charged are unable to cross cell membranes, the use of nanocarriers allows for their increased cellular uptake [113]. One example is the use of chitosan NPs, which, being positively charged, fulfill the role well [114]. The use of dendrimers has already been tested for the therapy of this deadly cancer, and reduced proliferation in vitro and reduced metastasis in vivo were described [115]. As above mentioned, AuNPs do not appear to show allergic or adverse effects and have an excellent ability to be manipulated. Indeed, their use can be associated with siRNAs that induce apoptosis in ovarian cancer cells [116]. The two molecules have been tested together to exploit their synergistic effect and to inhibit the causes of resistance to therapies. For example, it has been shown that the apoptosis of cancer cells can be increased by exploiting the effect of pro-apoptotic siRNAs with doxorubicin [117].

## 7. Conclusions

**Figure 1:**
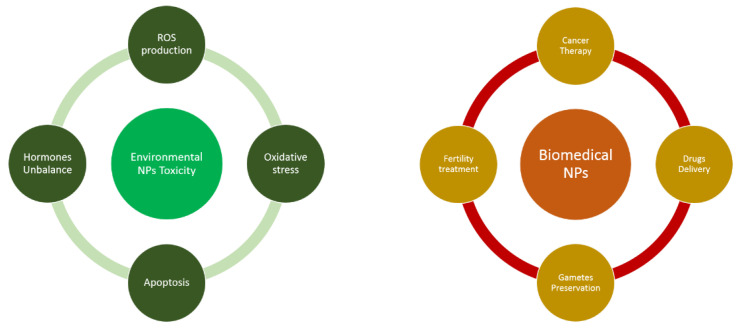
Toxicity and biomedical applications of environmental and artificial NPs.

The fact that NPs can harm biological systems but can be used for innovative therapies capable of conferring health benefits highlights the two sides of a coin (Figure 1). To lessen their toxic side effects, it’s necessary to reduce environmental pollution and to increase our knowledge of the pathways by which NPs enter human body to modulate cell metabolism. At the same time, the production of NPs able to select specific organs or cells will be a non-invasive option for successful molecular therapy of many human pathologies as well as of genetic anomalies, especially of embryos or oocytes. In this context, the development of new treatments of pathological reproductive conditions and the most frequent complications of pregnancy are needed and are of great importance for women’s health. Different coatings able to reduce toxic effects (e.g., albumin, polyethylene glycol, aspartic acid) are under investigation to facilitate their penetration into cells and to increase NP stability and targeted distribution in human body. From inorganic and organic particles, technologies are also moving toward new chemical forms such as lipid NPs. One of the most recent applications is in vaccines. With the COVID-19 outbreak and the licensing of mRNA-based vaccines, nanostructural technologies have been used to transport mRNA. These correspond to liposomes, nanostructures characterized by an inhomogeneous mixture of lipid components: neutral or charged lipids that promote the entry of nucleic material by electrostatic interaction, cholesterol that decreases permeability and increases stability, PEG-lipids that hinder binding to nonspecific proteins and helper lipids that facilitate their fusion with target membranes [118,119,120]. Thus, distinguishing the applications of nanomedicine from nanotoxicology becomes critical, and understanding the cut-off point between biomedical application and adverse effects will allow us to improve possible therapeutic applications: only by combining diagnostic testing and prevention will it be possible to improve both sides of this coin.
